# Electroconvulsive therapy-induced motor seizure length trajectories and their association with clinical remission: a retrospective cohort study across diagnoses

**DOI:** 10.3389/fpsyt.2026.1706968

**Published:** 2026-02-20

**Authors:** Eduardo Tedeschi, Mauricio S. Hoffmann, Pedro V. S. Magalhães

**Affiliations:** 1 Programa de Pós-Graduação em Psiquiatria e Ciências do Comportamento, Faculdade de Medicina, Centro de Pesquisa Clínica, Hospital de Clínicas de Porto Alegre, Universidade Federal do Rio Grande do Sul, Porto Alegre, Brazil; 2 Departamento de Neuropsiquiatria, Universidade Federal de Santa Maria, Santa Maria, Brazil; 3 Care Policy and Evaluation Centre, London School of Economics and Political Science, London, United Kingdom

**Keywords:** electroconvulsive therapy, growth trajectories, longitudinal study, motor seizure length, remission

## Abstract

**Background:**

Electroconvulsive therapy (ECT) is a well-established treatment for severe psychiatric conditions, yet optimal process markers for predicting therapeutic response remain under investigation. Historically, motor seizure length has been considered a factor in ECT efficacy, but the relationship between seizure length and treatment response remains controversial.

**Methods:**

We conducted a retrospective cohort study that included 331 inpatients who received ECT for acute psychiatric conditions between 2009 and 2015. Growth Mixture Modeling (GMM) was used to identify seizure length trajectories, while Latent Growth Curve Modeling (LGCM) examined predictors of changes. Clinical remission was assessed via the Clinical Global Impression (CGI) scale pre- and post-treatment.

**Results:**

A single decreasing trajectory of seizure length over ECT sessions best fit the data. Baseline seizure length and session frequency were significantly associated with changes in seizure length. However, neither seizure length nor its reduction correlated with clinical remission.

**Conclusion:**

Motor seizure length decreased across ECT sessions but was not associated with clinical remission in this study. Limitations include the single-site, retrospective design and the absence of EEG-based seizure measures, which may limit generalizability. These findings challenge the utility of seizure length as a predictive marker and underscore the need for more precise indicators to tailor ECT interventions.

## 1 Introduction

Electroconvulsive therapy (ECT) remains a vital psychiatric treatment, widely used in many severe psychiatric conditions where other therapeutic modalities have failed or were not viable (1). Since its introduction in the 1930s, ECT has evolved significantly in technique, safety, and acceptance, although still a target of controversy and stigmatization (2). Robust studies demonstrate its efficacy in disorders such as major depression, bipolar disorder, schizophrenia, and catatonic states, notable for the rapidity of response (3).

In addition to being able to elicit seizure activity, the duration of the convulsive seizure during ECT, often observed between 20 and 60 seconds, has historically been considered a criterion to evaluate the effectiveness of the treatment (4). The hypothesis that longer seizures are associated with more effective therapeutic outcomes was supported by studies that demonstrated a positive correlation between seizure length and clinical improvement in patients with major depression (5). In turn, Sackeim’s anticonvulsant hypothesis posits that the therapeutic effects of ECT arise from neurophysiological changes that reduce neuronal excitability and reorganize neural circuits (6). As such, one possibility is that the seizure threshold will increase with repeat treatment, thus reducing seizure length. In that case, a shortening motor seizure length, as a proxy for increasing the seizure threshold, could be a marker of response to ECT. Furthermore, there is growing discussion regarding the possibility of distinct patterns of seizure length across ECT sessions, which may reflect individual patient tendencies and variability during treatment (7). Recent studies suggest that seizure quality measured by EEG parameters might be a more precise indicator of therapeutic efficacy than length alone (8). Consequently, the focus has shifted towards understanding how these patterns and interindividual variability can inform the personalization and optimization of treatment.

In summary, the controversy surrounding seizure duration in ECT underscores the need to investigate not only average seizure length but also the variability across sessions and how these factors correlate with clinical outcomes. This approach may provide deeper insights into the personalization of treatment and a more comprehensive understanding of the mechanisms through which ECT exerts its therapeutic effects. In this study, we report on the behavior of seizure duration in the same individual throughout a course of ECT across diagnostic categories in a retrospective inpatient cohort. We then investigate the relationship between seizure length and clinical remission, considering its relationship with individual parameters such as the use of medications, categorical diagnosis, diagnostic staging models, and intrasession data, including pulse and frequency. To evaluate seizure length and its trajectories during the course of ECT, we used the Growth Mixture Model (GMM), which is effective in capturing individual variations over time. The study by Cheng et al. demonstrated the applicability of GMM in mental health analyses, particularly in older adults, highlighting the importance of identifying depression and anxiety trajectories (9). Additionally, Stlhofer (10) applied growth modeling to examine the dynamics of psychological factors, such as depressive and anxiety symptoms, among adolescents, reinforcing the value of this approach for understanding changes over time. GMM allowed us to test the hypothesis of different treatment response trajectories in ECT, as discussed by (11), who investigated material hardship trajectories and their relationship with mental health. Our primary hypotheses were that decreases in motor seizure length that happen during a course of ECT are a marker of clinical improvement and that variations in seizure duration across sessions may differ between individuals, serving as a potential predictor of treatment response. We believe that analyzing this variability, in addition to the absolute seizure duration, may provide important indicators of ECT efficacy and aid in personalizing therapeutic interventions.

## 2 Materials and methods

### 2.1 Participants

We included every adult inpatient who underwent ECT for an acute (i.e., not maintenance) indication at Hospital de Clínicas de Porto Alegre from January 2009 to December 2015 (12). The study was approved by the Ethics Committee of Hospital de Clínicas de Porto Alegre.

### 2.2 Electroconvulsive procedure

Sessions were conducted at the outpatient surgical center thrice weekly. A general anesthetic (thiopental, 3mg/kg) and muscle relaxant (succinylcholine 0.75-1mg/kg) were routinely administered, as per institution protocol. The service standard was high-dose and electrode placement was right unilateral, with the titration method used to determine stimulus dose in the first session. From the second session onwards, the stimulus charge was set at 6 times the seizure threshold. Stimulation dose was not routinely adjusted to compensate for changes in motor seizure duration across the treatment course. Three ECT devices were in use during this period, the MECTA 5000 M (between 2009 and 2013), the MECTA 5000 Q (between July 2013 to January 2014) and the MECTA 4000 Q (from January 2014 onward) (MECTA Corporation, Tualatin OR, USA). This reflects the replacement of MECTA models by the service within the same manufacturer line (MECTA 5000M, 5000Q, and 4000Q). Because each device model has different technical specifications, the stimulation parameters (amplitude, pulse width, and frequency) were adjusted as required to achieve the target charge. No relevant differences in output parameters were expected to influence seizure duration.

The treating psychiatrist observed and measured the seizure length in seconds from the onset of clonic convulsions; the cuff method was used to block the distribution of the muscle relaxant to the forearm. Patients can be stimulated more than once, but here we use only the first stimulus for the reported analysis (13). For each recorded session, we extracted data on ECT device parameters. We recorded the dosage of thiopental and succinylcholine and whether the patient received a beta-blocker, and medications used intrasession.

The medical records of the patients were inspected for gender, age and ethnicity, as well as for medications used, the day before the procedure and primary ICD-10 diagnosis at discharge. For this report, we grouped the diagnoses as depression (unipolar or bipolar), mania, psychotic disorders (schizophrenia, schizoaffective disorder, and delusional disorder), and other diagnoses as a residual category. The ‘Other diagnoses’ group included anxiety, substance use, organic, and movement disorders, which were combined due to limited sample sizes. This heterogeneity is acknowledged as a limitation. Independent raters blinded to ECT session data also established a Clinical Global Impression (CGI) score from the medical records before and immediately after the ECT series. We further characterized complete remission for the main outcomes as a CGI score of 1. The scale is composed of items ranging from 0 to 7, with 0 being *not assessed*, 1 *not ill*, and 7 *extremely severe.* The CGI was used because of its ease of use by experienced raters and the transdiagnostic approach employed here (14, 15).

### 2.3 Growth mixture modeling

Seizure length trajectories were estimated with GMM (16). GMM tested the smallest number of trajectory classes that captured the most variance at the individual level. It also allowed random variability around the mean trajectories within each trajectory class. For each class, we estimated the intercept, the slope and the quadratic function of the slope to estimate if changes were accelerating or slowing down over time. We compared three models (one to three classes) to determine the model that best fits the data. For the comparison, we used the Log-Likelihood, Entropy, and the Akaike information criterion (AIC). Solutions were compared using the Likelihood Ratio Test (LRT), with p-values below 0.05 indicating the statistical superiority of the compared class. Furthermore, we evaluated the content and theoretical meaningfulness of the classes in the various solutions by examining the sample size in each solution and parameter statistical significance. The best-fitting model associated with the examination of sample size in each class determined the classes to be used for further analyses. We conducted a sensitivity analysis restricted to remitted patients to evaluate whether responders exhibited distinct seizure-length trajectories. This subgroup GMM allowed us to assess potential within-group heterogeneity that might not be detectable in the full sample.

To visualize the relationship between seizure-length trajectories and remission, we plotted seizure length across sessions for all subjects and stratified by remission status (CGI remitters *vs*. non-remitters).

### 2.4 Latent growth curve model

We applied LGCM to test a model that estimated the growth of seizure length by partitioning the intercept, or average rate of seizure length change (i.e., tendency), and the slope, or the rate of seizure length change over time, for each trajectory class found in GMM. After that, we evaluated the variables that are associated with seizure length growth.

In LGCM, indicators (e.g., seizure length in seconds) could be specified in the measurement model as latent variables in the structural part of the model (17, 18). LGCMs could evaluate growth underlying the observed seizure length at each time-point and estimate which variables would be associated with the variability in the rate of change of psychiatric diagnosis over time (17, 18). We first specified an unconditional model (e.g. models without observed predictors) to evaluate if the growth model fits the data and estimate the variability of growth factors (intercept and slope) of seizure length. Significant slope variance suggested inter-individual differences surrounding the average rate of change and would justify investigating predictors of this variability through conditional models. Thus, in the second stage, we specified a conditional model including our main predictor, age, CGI, anticonvulsants, beta-blockers, session frequency, train duration and thiopental dose. Covariates were selected based on previous studies reporting associations with seizure quality or duration (e.g., use of beta-blockers, train duration, and anesthetic dose), rather than exploratory inclusion.

The LGCM specification included continuous latent variables as growth indicators (intercept and slope) and the residual variance was set to be invariant over time. The covariance between a person’s average (intercept) was set to correlate with the rate of change (slope). The intercept was specified by setting the factor loadings of seizure length measurements to 1 for all time points. The slope was specified by setting the factor loadings to be zero at the first time point, and subsequently adding 1 to the time intervals, as similar intervals were given within each ECT session. For the conditional model, latent intercept and slope were regressed on the predictors, which were treated as time-invariant covariates as they did not vary significantly over time (e.g., medication dose, etc.). We presented standardized results in the original outcome’s metric (i.e., seconds).

LGCM analyses were performed using the robust maximum-likelihood estimator (MLR) and full information maximum likelihood to deal with missing data. The evaluation of the model’s fit was conducted using the χ^2^ goodness-of-fit statistic (the model was accepted if p-value ≥0.05), Root Mean Square Error of Approximation (RMSEA<0.06), the Comparative Fit Index (CFI>0.90) and Standardized Root Mean Square Residual (<0.08) (19). We used scaled fit statistics for all LGCM analyses.

To reduce attrition bias, all LGCM were adjusted using inverse probability weighting (IPW) (20). *Probit* regression models were used to estimate baseline variables that predicted the propensity of attrition at session 8. Baseline variables were selected based on a comparison of their differences at sessions 2 and 8. The predicted probabilities of attrition were used to estimate the IPW. According to these scores, complete cases were weighted by the inverse of their probability of being a complete case (20). All LGCM included the IPW as sampling weight to reduce attrition bias.

GMM was carried out using the “lcmm” package (“Estimation of Extended Mixed Models Using Latent Classes and Latent Processes: The R Package lcmm (Journal of Statistical Software,” n.d.) and LGCMs was carried out using the lavaan package (21). All analyses were conducted in R [Version 4.2.1] (22) and RStudio [Version 2022.12.0].

## 3 Results

**Figure 1:**
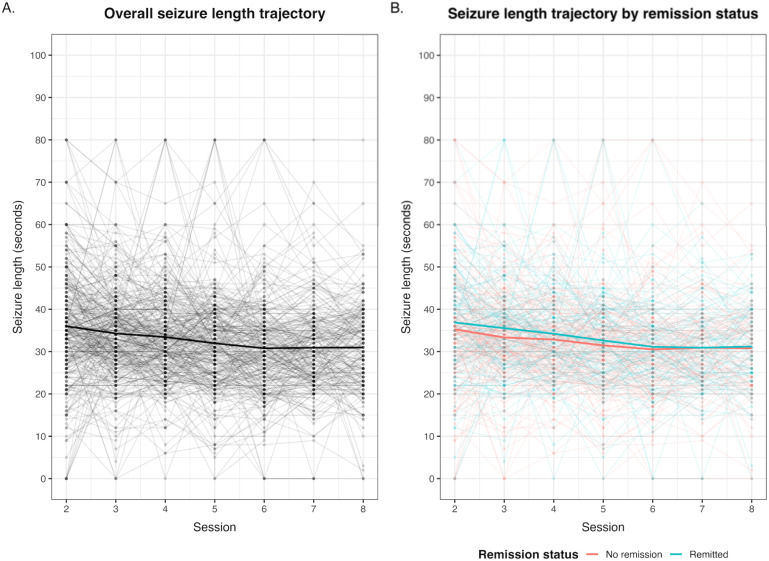
Seizure length trajectories across ECT sessions. **(A)** Individual seizure durations (light gray) and the mean trajectory (black) across all patients. **(B)** Seizure trajectories stratified by remission status (CGI = 1 vs. CGI > 1), with individual paths in faded colors and mean trajectories in red (no remission) and blue (remission).

The sample consisted of 331 individuals who underwent a second ECT session, of which 210 (63.4%) completed at least 8 sessions (see **Table 1** for demographic and clinical characteristics of the participants). The **Supplementary Material** also provides a descriptive comparison between patients who completed at least eight ECT sessions and those with shorter treatment courses (**Figure 1A**). Patients had a median change in CGI of 4 points, with a little over half (53%) having total symptom remission.

**Table 1:** Clinical characteristics of patients who received electroconvulsive therapy.

Variable	Median (IQR)	N	%
Age	47 (31 - 60)		
Motor seizure length	30 (25 - 35)		
Male sex		103	47,90%
Primary diagnosis	Depressive episode	100	46,50%
	Psychosis	68	31,60%
	Mania	31	14,40%
	Other	16	7,40%
Antidepressant use		64	30,30%
Lithium use		4	1,90%
Anticonvulsant use		17	8,10%
Antipsychotic use		183	86,70%
Benzodiazepine use		39	18,50%
Betablocker use		42	19,50%

We estimated three seizure length trajectory models. The first model presented one trajectory in which all participants belonged to a single class (named decreasing seizure length), with an intercept (i.e., average seizure length) of 39.9s (p<0.001) with an accelerated (B = 0.123, p=0.030) negative slope (i.e., seizure length becoming shorter over time) of -2.184s (p<0.001) (**Figure 1B**; **Supplementary Table S1**). The second model estimated a second class of individuals (named “increasing-decreasing”, n=5) that presented a statistically insignificant trajectory of a lower intercept, high slope, which slowed down over time (**Supplementary Table S1**). The third model presented the above-mentioned trajectories, dividing the second trajectory into two, one (n=4) as above, with a significant seizure length increase and decelerating trajectory, and one trajectory (“rapid increase-decrease”, n=1) (**Supplementary Table S1**).

Model comparison indicated that participants were better classified into three classes, as evidenced by likelihood, Entropy, AIC and LRT comparisons (**Supplementary Table S1**). However, the 2- and 3-class models marginally improved fit indices (ΔAIC ≈ –16; entropy ≈ 99%) and these additional classes contained only 4 and 1 participants, respectively. Moreover, their intercept parameters were not statistically significant, suggesting that they did not represent stable or interpretable seizure-length trajectories. The one-class model showed a highly significant intercept and slope, indicating a robust decline in seizure duration across sessions. Considering both parsimony and interpretability, we therefore retained the one-class model for subsequent analyses. Considering one-class solution (decreasing seizure length over time), this pattern occurred in remitters and non-remitters alike, demonstrating that both groups present progressively declining trajectories, with no substantive differences between them.

Because a substantial proportion of patients achieved remission, we re-estimated the latent class mixed model restricted to remitted individuals (CGI = 1; n = 146). In this subgroup, a 1-class solution best fit the data (AIC = 6477.52; entropy = 100%), with seizure duration starting around 41 seconds and showing a gradual decline over sessions, mirroring the pattern observed in the full sample (Figure). A 2-class solution yielded a very small second class (n = 4; 2.7%) and did not significantly improve model fit over the 1-class model (LRT = 6.02, p = 0.20; AIC = 6479.51; entropy = 92.7%). A 3-class model did not converge. These findings indicate that even among responders, seizure length follows a single, slowly decreasing trajectory with no evidence of clinically meaningful latent subgroups.

For estimating LGCM, we first derived IPW, which is described in **Supplementary Table S2**. The seizure length growth model presented an excellent fit to the data (χ^2^ = 27.423, p=0.549; RMSEA = 0.000, 90%CI= 0.000 to 0.036; CFI = 1.000; SRMR = 0.073). The intercept was estimated at 34.55s (z-value = 65.287, p<0.001) and the slope at -0.738s (z-value = -4.942, p<0.001), with a nonsignificant correlation of -3.825s (z-value = -1.917, p=0.055).

The conditional model (i.e., intercept and slope regressed on the predictors) also showed an excellent fit to the data, including additional predictors to explain the variation in seizure times and their changes over time. (χ^2^ = 75.684, p= 0.730; RMSEA = 0.000, 90% CI = 0.000 to 0.023; CFI = 1.000; SRMR = 0.046). The intercept was estimated at 38.517s (z-value =10.217, p<0.001) and the slope at -1.394s (z-value =-1.269, p<0.001), with a nonsignificant correlation of -1.263s (z-value = –0.678, p=0.498). The predictors considered were: age, remission (CGI = 1), anticonvulsants, beta-blockers, repeated sessions, session frequency, train duration, and thiopental dose. The duration of seizures and the number of repeated sessions had significant impacts on the slope, indicating that longer durations and more repeated sessions were associated with a reduction in seizure duration over time (**Supplementary Table S2**). Additionally, anticonvulsants showed a significant effect on the slope (**Supplementary Table S2**). Specifically, there was no association between changes in motor seizure duration and remission.

## 4 Discussion

We provide here a detailed analysis of motor seizure length trajectories observed during a series of ECT sessions and their relationship to clinical efficacy. The most parsimonious model was characterized by a single slow decline in motor seizure length over the sessions in all subjects. Moreover, this trajectory can be modeled with two components of seizure length: an intercept (personal average or tendency) and a slope (widening or shortening seizure length above and beyond the average). We found that these components of seizure duration were related to some clinical parameters but not related to clinical remission. This observation contrasted with our initial hypothesis that seizure length would be associated with clinical improvement.

Our first test was aimed at examining whether there were different seizure length trajectories over time, which could help identify distinct groups with different clinical profiles. However, although information criteria suggested that multi-class solutions might provide a slightly better statistical fit, close inspection showed that the additional classes comprised only a few participants and lacked significant intercepts or slopes. These characteristics imply that the small classes likely captured idiosyncratic fluctuations rather than genuine subgroups with distinct clinical meaning. Retaining the one-class solution allowed us to model the main, statistically robust trend of progressive seizure shortening over treatment, avoiding over-fitting to random noise. This decision follows established recommendations in growth-mixture modeling to favor simpler models when extra classes are unstable or clinically uninterpretable.

The relevance of seizure length as a marker of ECT efficacy has been previously investigated in studies, such as Maletzky’s, which demonstrated a positive correlation between seizure duration and clinical improvement in patients with major depression (5). However, our findings challenge this perspective, as we found no significant association between seizure duration and symptom remission in a transdiagnostic sample. More recent findings, including those by de Arriba-Arnau et al, emphasize that electroencephalographic features such as ictal amplitude, postictal suppression, and coherence may provide more accurate indicators of therapeutic efficacy than motor seizure length alone (8).

From a mechanistic perspective, ECT is known to modulate large-scale neural circuits, reduce hyperconnectivity in networks implicated in affective and psychotic disorders, and enhance neuroplasticity and neurogenesis (23, 24). These neurobiological processes are not necessarily captured by motor seizure length, reinforcing the need for multimodal monitoring. The progressive reduction in seizure duration across sessions likely reflects a physiological increase in seizure threshold, a well-documented phenomenon in the literature. However, our findings suggest that this reduction is not a sufficient condition for clinical improvement.

This study had several limitations that should be considered when interpreting the results. First, the sample consisted of inpatients from a single hospital, which might have limited the generalizability of the findings to other populations of less severely affected patients or clinical settings. This is a transdiagnostic sample and, although we did attempt to examine interactions with primary diagnoses, the study may have been underpowered to detect such differences. Although we employed robust statistical methods to analyze seizure duration trajectories, the possibility of unmeasured variables influencing the results could not be ruled out. Retrospective studies are also inherently limited by having their data collected for purposes other than research. Second, while we explored multi-class solutions, the very small and statistically non-significant subgroups precluded stable estimation or meaningful clinical interpretation. Because electrode placement was predominantly unilateral across diagnostic categories, the generalizability of diagnosis-specific efficacy interpretations may be limited. Future studies with larger samples may clarify whether subtle subgroups of seizure-length trajectories exist across diagnostic categories. Third, the absence of a detailed analysis of neurophysiological seizure parameters, such as EEG wave morphology, which might have been more precise indicators of seizure quality. As such, the lack of association between motor seizure duration and remission does not necessarily refute the anticonvulsant hypothesis of ECT efficacy (6), as seizure threshold was not directly measured in our study. Motor seizure duration is easy to observe but may not fully capture cortical seizure quality. Gilving (25) reported an association between EEG seizure duration during the first ECT session and remission in unipolar major depression. However, their analysis did not examine longitudinal trajectories across the treatment course. EEG duration reflects cortical seizure expression more directly, whereas motor duration may be a less reliable proxy. The present findings, therefore, remain consistent with this evolving literature: longitudinal changes in motor seizure duration do not appear to index treatment efficacy, even though baseline EEG duration may carry prognostic information under specific conditions. Recent work has demonstrated that motor and EEG seizure duration are only modestly correlated and often diverge substantially when used to classify seizure adequacy (26). These discrepancies indicate that motor seizure duration may be an imprecise proxy for cortical seizure dynamics and help explain why longitudinal motor duration trajectories in our cohort were not associated with clinical remission. EEG-derived measures such as ictal amplitude, recruitment, coherence, and postictal suppression have shown stronger associations with treatment response than motor seizure duration (27).

Furthermore, the study did not assess the impact of different ECT techniques and devices, which could have influenced session outcomes. The remission rate observed in our cohort (53%) falls within the range reported in contemporary real-world ECT cohorts, with variations likely reflecting differences in diagnostic composition, baseline severity, and remission criteria (28).

## 5 Conclusion

Our results indicated that the most common trajectory of seizure duration was a slow decline over ECT sessions. Moreover, seizure length components (intercept and slope) had no significant association with symptom remission across diagnoses. The findings in this report question the utility of motor seizure length as a contributor to seizure quality and as a mediator of ECT efficacy, although, in clinical practice, motor seizure duration remains important for procedural monitoring and safety.

## Data Availability

The raw data supporting the conclusions of this article will be made available by the authors, without undue reservation.
